# From theory to practice: Monetary policy transmission and bank risk dynamics

**DOI:** 10.1371/journal.pone.0299209

**Published:** 2024-04-18

**Authors:** Zheng Zhang, Joel Clovis, Peter Moffatt, Wenxue Wang

**Affiliations:** 1 School of Finance, Shandong Technology and Business University, Yantai, PRC; 2 Nottingham Business School, Nottingham Trent University, Nottingham, United Kingdom; 3 School of Economics, University of East Anglia, Norwich, United Kingdom; Universiti Malaysia Sabah, MALAYSIA

## Abstract

This paper investigates the relationship between monetary policy and bank risk-taking by introducing a model wherein banks expend a level of costly monitoring effort to select low-risk projects, thereby reducing the risk associated with the loans they grant. The impact of monetary policy on bank risk-taking is examined through both theoretical models and empirical analysis. The paper compares theoretical models with different assumptions, revealing an unambiguous negative effect without the assumption of limited liability for banks, and an ambiguous effect with the assumption of limited liability for banks, influenced by the equity ratio. The empirical model employs unique quarterly data comprising balance sheet information for top-listed banks in the U.S. banking system from 2000 to 2017. The findings indicate that low-interest rates contribute to an increase in bank risk-taking. Moreover, this effect is more pronounced after the financial crisis and weaker before the crisis. Additionally, the impact is evident for undercapitalized banks and more substantial for those financed with a higher proportion of equity.

## 1 Introduction

Taking risk is an integral part of the banking business, they had to try managing risk since the emergence of the banking industry. Until now, avoiding excessive risk exposures is still one of the principal rules for bank risk management. The run-up to the global financial crisis of 2007 to 2008 was marked by what became known as excessive risk-taking in the financial sectors, and the banking sector in particular [1–3]. As a result of this accumulation of risks, it led to severe systemic problems and the eventual collapse of many financial institutions, when the crisis finally occurred. Thereafter the whole world witnessed the collapse of some major financial institutions during the financial crisis, it also helped the policy-makers to recognize that the stability of financial systems should be at the forefront of policy. Hence a better understanding of the causes of bank failures, especially the aspect of bank risk-taking, is an essential way to help the banks, as well as the worst affected countries, to avoid the significant welfare losses in the future.

The main attention of most researchers, before the crisis, was on the impact of monetary policy on credit quantity in both macroeconomic theoretical models and empirical models (such as the literature on the bank lending channels), rather than the impact of monetary policy on bank risk-taking [4, 5]. Following the crisis, increasingly more economists, policymakers and bankers debated whether a low interest rate spurs risk-taking by banks. According to Jiménez et al. [2], from the start of the recent crisis, the market commentators argued that during the long period of quite low interest rate, bank had softened their lending standards and taken on excessive risk. Meanwhile, some other researchers [1, 3, 6, 7] held the opinion that low long-term interest rates and other factors were the cause of the current financial crisis. Despite that, at the same time, investors, firms, and other market participants still requested central banks to reduce the monetary policy rate to improve their financial situation, which made the whole financial market even worse.

Although it is not difficult to recognize that the low interest rate was not the main cause of the recent crisis, there is still no doubt that a low interest rate can contribute to the building up of the crisis. Due to the recent COVID-19 pandemic, lots of central banks continue to resort a lot easing monetary policy to stimulate the economy. According to Altunbas et al. [8], there are mainly two ways in which a low interest rate may have an influence on bank risk-taking. First, low interest rates can affect valuations, incomes, and cash flows. All these factors in turn can influence how banks measure risk [9, 10]. We call this the value effect.

Second, a low interest rate means low returns on general investments, such as government risk free securities, and it also means a low inducement to some investors. It may increase the incentives for banks and other financial institutions to search for some high growing (more risk) investments, to get a higher target of return [7, 11]. Generally, the risky assets will be more attractive if the short-term interest rate is low. As a result, investors may search for yield by financial intermediaries in the short run. This will cause both serious agency problems and a strong reliance on short term funding. Hence, the low interest rate may spur bank risk-taking. This is known as the search-for-yield effect. The sustained low interest rates stimulate the boom of global capital market and credit market, and it encourages financial institutions to take too much risk under higher leverage.

At the same time, we believe low interest rate induces the third effect, that is it may reduce the degree of risk aversion of banks and other institutions (generally the risk aversion of the shareholders and the managers), which can also induce the financial imbalance. As a result, many banks will ignore a certain extent of risk of themselves. It is not clear that the financial agents are even aware of this effect. It is this part of monetary transmission mechanism that has been termed the risk-taking channel of monetary policy by Borio and Zhu [10], which highlights the influence of monetary policy on the risk perceptions and the risk-tolerance by economic agents. Therefore, the third effect is termed as risk-taking-channel effect.

So, it is significantly important that we analyse one of the main causes of the recent financial crisis from a bank risk-taking perspective. Meanwhile, studying this topic will also complement the current theory, because there is not enough literature and the theoretical foundations for this topic have not been sufficiently discussed yet. The macroeconomics models have generally looked more at the quantity of credit rather than the credit’s quality, such as, the impact of short-term interest rates on the aggregate volume of credit in the economy [4, 5, 12]. The papers which thought about risk, have mainly studied how the monetary policy rate changes the level of risk on the borrowers’ side, rather than the side of financial institutions’ risk attitude [13, 14]. According to Laeven et al. [15], a large amount of banking literature has focused on the excessive risk-taking by financial intermediaries which operate under limited liability and asymmetric information, while most of these literatures ignored monetary policy. Thus, this is a potential gap, which this paper will try to fill.

The regulators or the banks themselves can access various instruments or methods in attempting to reduce the probability of a bank failure. One such instrument and method is that of effective monitoring. This paper introduces a model about banks spending a level of costly monitoring effort to choose low risk projects which the banks can afford and so reduce the risk of the loan the bank granted. This model will be used to analyse the effect of monetary policy on bank risk-taking in two different ways: with the assumption of limited liability of the bank in case of failure; and without the assumption of limited liability of the bank in case of failure.

The remainder of this paper will be organized as follows. The next section briefly introduces the current research in both related theoretical and empirical papers. Section 3 compares two theoretical models with different assumptions and analyse the relationship between monetary policy and bank risk-taking. Section 4 discusses the empirical analysis based on the theoretical models. Section 5 concludes.

## 2 Literature: Theory and evidence

In the financial market, low interest rate (policy rate), determines the market risk free rate and can influence bank risk-taking in several different ways. Three broad approaches have been used to explain the connection between them. These three approaches are consistent with the three effects introduced in the first section. The first approach to influence bank risk-taking decision by low interest rate is through the value effect. It focuses on the impact of low interest rate on valuations, incomes, and cash flows and measured risk [8]. All those factors will have a direct effect on the banks’ risk-taking decision. If the interest rate remains low in the long period, the value of asset and collateral will be affected. What is more, it will also affect the banks’ estimation of the probability of default, the loss given default, and the market volatility. Meanwhile, if the managers of banks that think the low interest rate will still last for a long period and the financial market will keep in a positive condition, it will reduce the risk aversion of banks. So, easy monetary conditions may increase the banks’ risk tolerance (the search-for-yield effect). This is quite close to the financial accelerator, in which increases in collateral values reduce borrowing constraints [14]. However, others maintain that the risk-taking channel is distinct but complementary to the financial accelerator, because it focuses on the amplification mechanisms, which allow relatively small shocks to propagate through the financial, due to financing frictions in the lending sector [9].

Secondly, the second channel which is the ‘search-for-yield effect’, as described by Rajan [7] is not new. It also appeared in the Global Unbalance literature [16, 17] and in the Feldstein Horioka puzzle literature [18, 19]. It suggests that low interest rate may tempt the asset managers and single investor to take on more risks. They may blindly pursue return and ignore some potential risks and uncertain factors. When the interest rate is high, banks can invest in riskless assets to obtain the return. However, they have to invest in some risk assets to pay back their debt, when the interest rate is low. And according to Shleifer and Vishny [20] and Brunnermeier and Nagel [21], there is a quite similar mechanism which might be in place when private investors use short term return as a way to judge the managers’ competence and withdraw funds after some poor performance.

The third way, risk-taking may also be affected by the risk-taking-channel effect. Such as, the communication policies of a central bank and the characteristics of the policymakers’ reaction function, comes from the principal agent problem, in the form of moral hazard [8]. If a central bank predicts its own future policy decisions to a quite high degree, the market uncertainty would be reduced, and banks would like to take many more risks. As a result, the agents and investors will believe that the central bank will ease the bad outcome of monetary policy. Diamond and Rajan [22] stressed that the monetary policy should be kept tighter than the degree of the current economic conditions. And it will reduce the probability of banks taking on liquidity risk. Another example of the risk-taking-channel effect can be shown in the case of ‘The Greenspan Put’. In this situation, the central bank will generally take powerful measures to control the state economy and help financial stability, when the economic downturn occurs. The expectation of a bank which the central banks will help to contribute the financial stability, may lead banks to take more risk [23]. So, in this case, banks can increase their yield by taking more risks. Even if banks default due to the excessive risk-taking, they will believe that the central bank is still willing to support them to help the state economy and financial stability. Then, banks risk-taking can be affected by the central bank’s behaviour to the economic situation. However, this will also lead to a moral hazard, because banks may always believe that the central bank is willing to remedy for their excessive risk-taking.

Generally, the first two channels focus on the monetary policy environment, while the last one focuses on the response function of central banks. Alongside the theoretical evidence, there is an increasing volume of empirical literature attempting to test the link between monetary policy and bank risk-taking.

Jiménez et al. [2] used the microdata of the Spanish Credit Register from 1984 to 2006 to test whether the stance of monetary policy has an impact on the level of individual bank loans. They identified the effect of monetary policy on credit risk-taking with an exhaustive credit register of loan applications and contracts. They found that the high bank risk-taking comes with a period of easy monetary policy, which supports the search-for-yield effect. And due to higher collateral values and the ‘search for yield’, bank would like to grant a riskier loan and soften their lending standards (‘the risk-taking-channel effect’). In this case, banks will lend more to borrowers who have a bad credit history and borrowers with more uncertain prospects. Similarly, Maddaloni and Peydró [24] used the lending standard to study the determinant of banks’ lending standards in the Euro Zone’s banking. They stressed the impact of low interest rate on lending standards. Altunbas et al. [8] found that low interest rate will increase the lending risk-taking of banks, by using the data of listed banks in the European Union and the United States,

It was examined by researchers whether the risk-taking channel works on both the quantity of new loans and their interest rates, and they found that when the interest rate is low, banks increased the number of new risk loans [25]. What is more, they also reduce the rates, which they charge to the borrowers compared with the rates, which they charge to the less risk borrowers. The same investigators [26] studied the risk-taking channel of monetary policy in Bolivia. They found that “a lower policy rate spurs the granting of riskier loans, to borrowers with worse credit histories, lower ex-ante internal ratings, and weaker ex-post performance”.

## 3 Theoretical models

In this part, we will talk about a theoretical model, with two different assumptions: one is under the assumption of limited liability of the bank in case of failure, and the other one is without the assumption of limited liability of the bank in case of failure.

### 3.1 Theoretical model 1

#### 3.1.1 Assumptions and model framework

Consider a simple model, with two parties: firms (borrowers), and banks (lenders), in the financial market. These two parties can be described as follows:

Firms (Loan Borrowers): Firms can decide to invest in projects with different risk levels. Because each firm also has an external financial demand in the investment activity, they need to apply for a loan, which is supplied by banks. The firms or the projects a firm processing, can be graded according to their risk level. We assume that, a firm will have more chances to success, if the bank who grants loans to this firm pays more monitoring effort on it. That is because, for example, bank’s monitoring can help to reduce the agency problem between the shareholders and the managers of the firm, and as a result, the monitoring behaviour of banks can reduce the risk of the firm and increase the firm’s value. Another way of thinking about the reason why bank monitoring can improve firms’ performance is that banks can help firms in better behaviour according to the information banks observe. To be more specific, a firm with better performance and better behaviour means that it will have a higher probability to avoid failing and a higher probability of having the ability to repay the loan. What is more, banks’ financial expertise can also help firms improve their expected value [27–31]. For example, banks’ financial expertise can improve firm’s probability to success, and then the firm expected value will increase. Meanwhile, a more professional firm will also increase the confidence of the firms’ shareholders and investors, who will evaluate the firm highly, and increase the share price of this firm. As a result, the expected value of the firm with banks’ financial expertise will rise.

In this paper, firms’ demand function of loan is initially assumed as a linear function, which is negatively correlated with the loan rate charged by banks. This assumption is quite popular in the literature [32]. So, the loan demand function can be written as: *F*(*r*_*L*_) = *a*-*br*_*L*_, where *r*_*L*_ is the loan interest rate, charged by banks, both *a* and *b* are positive constants. The loan interest rates are different for different firms, and they are set according to the policy rate and banks’ evaluation of firms. We will relax this assumption and discuss two other cases in which two non-linear loan demand functions exist.

Banks who are the loan suppliers can invest in projects or firms with different risk levels. They can choose to monitor their loan portfolio to reduce their risk-taking and increase the probability of loan repayment. We assume that the banks monitoring also works in both good and bad states of the world economy. This is because banks will also have the ability to choose the acceptable firms or projects to invest, and to improve the probability of repayment, even in an extreme world economic condition. For example, in a bad state of the world, it is harder for most firms to be successful. But banks also need to grant loans to firms to get profits. They can use the monitoring effort to increase firms’ probability to succeed, even in a lower amount, compared with the case in an average or good economic environment. So, we use the bank monitoring effort as the proxy of bank risk-taking. The basic principle is that the more monitoring effort bank pays on its portfolio, the less risk will be borne by banks. This monitoring effort is costly and not contractible. The monitoring effort can also represent the probability of firm success and the loan repayment. As a consequence, banks can choose the level of monitoring to affect their profits of the investment to each firm. So, there should be an optimal monitoring level, under which banks can maximize their profits. The optimal bank’s monitoring level is not fixed, and it is different in different firms and projects. Banks finance themselves in two different ways. The first part is financed by debt, and the other part is funded by equity. For simplicity, we assume each bank will only lend to one firm, during the lending and borrowing process.

Regarding the bank monitoring, we assume banks have to choose a level of *q* (*q* ∈ [0,1]), which is the monitoring effort, to increase the probability of firm (the borrower) success and the loan repayment. The cost of bank monitoring is *cq*^2^ + *d* per unit borrowed by firms. Where, *c* is a positive constant, *d* is also a positive constant, which means the initial cost of the bank monitoring. The intensity [0, 1] of *q*, can also be interpreted as the probability of firms’ success, which is consistent with the assumption that the probability of firms’ success will increase, with a higher and higher monitoring level by banks. The convex cost function captures the idea that it will be increasingly difficult for banks to explore more and more information about the firm in which they invested. It means that banks need to pay more monitoring efforts to obtain the same amount of information about the firm, compared with the beginning stage. The convex bank monitoring cost function is supported by a large amount of literature [27, 30, 31, 33].

About the bank’s capital structure, we assume that the bank is financed with a portion of *k* in equity, at a cost of *r*_*E*_, and a portion of *1- k* in debt, at a cost of *r*_*D*_. Hence, *r*_*E*_ and *r*_*D*_ also mean the interest rate paid to the shareholders and the depositors. Both of them are positively related to the risk-free rate which is r^*^. This allows that monetary policy can change the cost of banks’ liabilities by changing the risk-free rate. Then, *r*_*E*_ and *r*_*D*_ can be expressed as: *r*_*D*_ = r^*^ + *α*, and *r*_*E*_ = r^*^ + *β*, where *α* ≥ 0, it can be regarded as the incentive to depositors; and *β* ≥ 0, it can be regarded as the incentive to equity investors. According to Laeven, et al. [15], in our model we assume that the premium on equity and debt, *α* and *β*, are independent of the policy rate, r^*^. That is consistent with our goal to isolate the effect of an exogenous change in the stance of monetary policy. However, this might be not consistent with the Modigliani and Miller theorem, because, from an asset pricing perspective, they are likely to be correlated. That might happen through the underlying common factors which may include both the risk premium and the risk-free rate. However, the results continue to hold as long as the within period correlation between them is sufficiently different from (positive) one. Generally, the equity premium as a spread over the risk-free rate can be used to explain the reason why *β* ≥ 0. On the other hand, banks always would like to pay a higher interest rate than the risk-free rate to the depositors, because they need to attract these investments from depositors with a higher return. If not, depositors will withdraw their savings, banks will not have enough funds to invest. They are broadly discussed in the literature [1, 15, 34–36].

The timing of our model can be explained as follows:

Stage 1, the policy rate is set;
Stage 2, banks choose the interest rate to charge on loan—*r*_*L*_, choose the interest rate paid to shareholders–*r*_*E*_, and depositors–*r*_*D*_, and also set the leverage level—*k*;
Stage 3, firms apply for the loan and borrow from banks at the rate of *r*_*L*_;
Stage 4, banks decide whether to grant the loan or not, and choose the monitoring effort *q*.

#### 3.1.2 Equilibrium bank monitoring

Given the amount of the factors in the above section, the bank can maximize the expected profits by choosing its optimal level of monitoring. To guarantee bank’s maximum expected profits, a negative second order condition must be satisfied. Here, we can write the bank’s expected profit as:

$$\Pi =\left[qr_{L}-(1-k)r_{D}-kr_{E}-C(q)\right]F\left(r_{L}\right)$$ (3.1.1)

*Where, Π is the bank’s expected profit*;

*q is the monitoring effort, or the probability of the loan repayment*;

*r_L_ is the interest rate charged by banks*;

*k is the portion of bank assets financed with equity*;

1*—k is the fraction of bank’s portfolio funded by deposits;*

*r_D_ is the interest rate paid to depositors by bank*;

*r_E_ is the interest rate paid to equity investors by bank*;

*C*(*q*) *is the cost function of bank monitoring*;

*F*(*r*_*L*_) *is the firms’ loan demand function*.

By substituting the expression functions of *r*_*D*_, *r*_*E*,_*C*(*q*), and *F*(*r*_*L*_) into the bank’s expected profit function, we can obtain a more detailed bank’s expected profit function:

$$\Pi =\left[qr_{L}-(1-k)\left(r^{*}+\alpha \right)-k\left(r^{*}+\beta \right)-\left(cq^{2}+d\right)\right]\left(a-br_{L}\right)$$ (3.1.2)

We can see from the bank expected profit function, the profit per unit lent equals the possible income of the loan (*qr*_*L*_), less the costs on both deposit and equity ((1 –*k*)(r^*^+*α*)+*k*(r^*^+*β*)), and minus the cost of bank monitoring (*cq*^2^ + *d*).

Eq (3.1.2) is a concave function of the bank monitoring effort. Consequently, there exists a maximum value of the bank’s expected profit, because the second order condition of bank’s expected profit respect to the monitoring effort is strictly negative. We can maximize the bank’s expected profit, by taking the first order condition of the bank expected profit respect to the bank monitoring effort, and letting the new function equal to zero. According to this, the optimal bank monitoring effort can be available. Then, it can be written as:

$$\frac{\partial \left[qr_{L}-(1-k)\left(r^{*}+\alpha \right)-k\left(r^{*}+\beta \right)-\left(cq^{2}+d\right)\right]}{\partial q}\left(a-br_{L}\right)=0$$ (3.1.3)

We can get the optimal bank monitoring level:

$$q^{*}=min\left\{\frac{r_{L}}{2c},1\right\}$$ (3.1.4)

We assume the value of $\frac{r_{L}}{2c}$ will always be not larger than one. So, the optimal value of bank monitoring effort will equal to $\frac{r_{L}}{2c}$. It means the relationship between the optimal bank monitoring level and the bank loan interest rate, which is positive: the higher the loan interest rate bank charges, the higher the monitoring effort bank pays. Therefore, a lower bank loan interest rate will reduce the monitoring effort, as a result, increase bank risk-taking. The intuition for this result is that a higher interest rate loan will increase the incentives for banks to monitor more on it to get a higher probability of receiving the loan repayment. That is because banks will value more on a higher interest rate loan.

Recalling the value effect, that we mentioned in previous sections, it is a simple mechanism based on the concept of expected profit in our model. By construction, an increase in the loan rate *r*_*L*_ increases expected profit for given monitoring effort *q*. Since the bank marginal profit increases linearly with the loan rate *r*_*L*_, the optimal monitoring effort q^*^ that the bank chooses to maximize profit (when taking the loan rate as given) will be higher with the higher the loan rate *r*_*L*_. With this intuition from the result, if one further postulate that the loan rate *r*_*L*_ is positively related to the policy rate r^*^, it follows that $\frac{\partial q^{*}}{\partial r^{*}}>0$. Therefore, by its nature, the value effect hinges on a generally positive relationship between the bank loan rate *r*_*L*_ and the monetary policy rate r^*^, and holds even when the bank does not choose the loan rate *r*_*L*_. These are also what we will test in the following parts.

Regarding the other two effects, the search-for-yield effect is a different mechanism based on the existence of market power. In fact, it only arises when the bank can choose the loan rate. When the bank is able to choose the loan rate *r*_*L*_, the profit-maximizing behaviour of the bank is similar to that of a monopolistic producer: the bank will raise the *r*_*L*_ (just like a monopolistic firm would raise the output price above the marginal cost) to maximize the pure rents created by a lower level of loans along with the demand schedule (just like the mark-up created by a lower level of output purchased by consumers under monopoly pricing). This means that when banks can set the loan rate, they will have an incentive to raise *r*_*L*_ to increase their yields; it will in turn reinforce the value effect via the endogenous response of the loan rate *r*_*L*_ set by banks to changes in the monetary policy rate r^*^. Based on our model, the risk-taking-channel effect will arise when the bank chooses the loan rate and there is limited liability. It is basically a moral hazard story: it tends to counteract the search-for-yield effect because limited liability gives the bank an incentive to reduce monitoring when the interest rate goes up, and vice versa.

In the following parts, we will discuss the relationship between bank monitoring effort level (bank risk-taking) and policy rate, when banks are facing a convex monitoring cost function, and firms have different loan demand functions.

#### 3.1.3 Linear loan demand function

Firstly, we consider the case where neither the loan interest rate (*r*_*L*_) nor the portion of bank capital *k* are determined by banks, for example, the regulator chooses the interest rate and the capital *k*. In Eq *(3.1.4)*, it means the optimal monitoring effort is fixed according to the level of loan interest rate (*r*_*L*_) and the coefficient of bank’s cost function (*c)*. Specifically, the higher loan interest rate set by the regulator will lead to a higher monitoring effort by banks, and the more the monitoring effort cost, the less monitoring effort banks will be willing to pay.

Then, we will analyse the situation that banks can choose the loan interest rate by themselves, while the capital k is still determined by the regulator. We can solve this relationship between bank monitoring effort and monetary policy by backwards induction. When we assume that monetary policy can change the cost of banks’ capital by changing the risk-free rate, so we just need to know the first order condition of optimal monitoring effort with respect to risk-free rate, which is $\frac{\partial q}{\partial r^{*}}$. In the Eq *(3.1.4)*, because the loan interest rate is a compound function of risk-free rate, we can get:

$$\frac{\partial q}{\partial r^{*}}=\frac{1}{2c}\left(\frac{\partial r_{L}}{\partial r^{*}}\right)$$ (3.1.5)

To obtain $\frac{\partial r_{L}}{\partial r^{*}}$, we substitute the optimal bank monitoring level q^*^ function *(3.1.4)*, into the bank’s expected profit function *(3.1.2)*:

$$\Pi =\left[\frac{r_{L}}{2c}r_{L}-(1-k)\left(r^{*}+\alpha \right)-k\left(r^{*}+\beta \right)-\left(c{\left(\frac{r_{L}}{2c}\right)}^{2}+d\right)\right]\left(a-br_{L}\right)$$

$$=\left[\frac{r_{L}^{2}}{4c}-r^{*}-(1-k)\alpha -k\beta -d\right]\left(a-br_{L}\right)$$ (3.1.6)

Assuming $\text{G}=\frac{\partial \Pi }{\partial r_{L}}=0$, by using the Implicit Function Theorem, we can get the second order condition of bank’s expect profit respect to the loan interest rate is:

$$\frac{\partial^{2}\Pi }{\partial r_{L}^{2}}=\frac{\partial G}{\partial r_{L}}=\frac{1}{2c}\left(a-3br_{L}\right)$$ (3.1.7)

The above second order condition Eq *(3.1.7)* will be positive, if $r_{L}<\frac{a}{3b}$, and it will be negative, when $r_{L}>\frac{a}{3b}$. However, we will only consider the range of $r_{L}>\frac{a}{3b}$, because only in this case, $\frac{\partial G}{\partial r_{L}}<0$, which guarantees bank can achieve the maximum profits consistently. So, in the following parts, we need to guarantee all the second order conditions of bank’s expected profit to be negative, when we talk about the bank’s maximum profit. According to this negative second order condition of bank’s expected profit respect to the bank loan interest rate, we can obtain a reasonable rang of loan interest rate. Here, we assume that the parameters *a* and *b*, always satisfy $r_{L}>\frac{a}{3b}$. And as result, $\frac{\partial^{2}\Pi }{\partial r_{L}^{2}}=\frac{\partial G}{\partial r_{L}}<0$, which means the bank’s maximum profit achieves.

Next, to analyse the relationship between the bank loan interest rate and the risk-free interest rate, we can now differentiate G with respect to r^*^. We will have the result:

$$\frac{\partial G}{\partial r^{*}}=b>0$$ (3.1.8)

$$\text{So},\frac{\partial r_{L}}{\partial r^{*}}=-\frac{\partial G/\partial r^{*}}{\partial G/\partial r_{L}}>0$$ (3.1.9)

Due to the Eq *(3.1.5)*, $\frac{\partial q}{\partial r^{*}}$ is positively correlated with $\frac{\partial r_{L}}{\partial r^{*}}$.


$$\text{Similarly},\frac{\partial q}{\partial r^{*}}>0$$ (3.1.10)

In the case that the banks can choose the loan interest rate by themselves, while the capital k is exogenous, the banks monitoring effort will increase (less risk taking by banks), with an increasing policy rate, and the banks monitoring effort will reduce (more risk taking by banks), when the policy rate decreases. Banks’ risk attitude will be unambiguous. The loan demand function tells us that the banks can choose a loan interest rate with an intensity ($0,\frac{a}{b}$). Specifically, banks can obtain their maximum profit, only in the case when the loan interest rate is set from $\frac{a}{3b}$ to $\frac{a}{b}$. In this range, it implies that the relationship between the amount of bank monitoring effort and the risk-free rate is unambiguously positive. It also means that the lower policy rates will spur the bank to take more risks. So, we can obtain the result:

**Proposition 1** *When the banks monitoring cost function is a convex function and the firms loan demand function is a linear function*, *if banks can decide the size of loan interest rate*, *r*_*L*_*, banks will always set it in the interval of $\left(\frac{a}{3b},\frac{a}{b}\right)$, and banks monitoring increases with monetary policy rate, $\frac{\partial q}{\partial r^{*}}>0$Banks take more risk with the monetary policy rate decreases.*

#### 3.1.4 Non-linear loan demand function

This section examines the effect of the alternative loan demand functions. We look at two different cases: first, the loan demand function is a concave function; while second, the loan demand function is a piecewise function.

*a. Concave loan demand function*. In the first case, a concave function, we can assume that the loan demand function is:

$$F\left(r_{L}\right)=a-br_{L}^{2},\;where,\;0<a\leq b,\;and\;0<r_{L}<\sqrt{\frac{a}{b}}.$$ (3.1.11)

**Fig 1:**
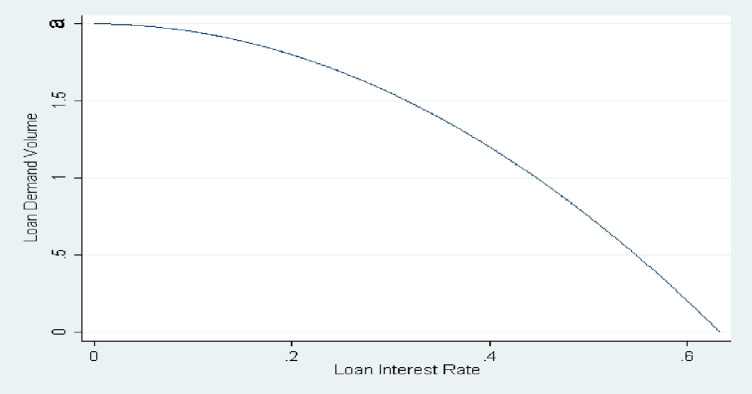
Concave loan demand function.

This concave loan demand function can be interpreted as: the intercept *a* is the firms’ maximum loan demand, when loan interest rate is zero. When $r_{L}=\sqrt{\frac{a}{b}}$, firms loan demand function will be zero, which means the loan interest rate $r_{L}=\sqrt{\frac{a}{b}}$, can be interpreted as either the maximum return on projects, or as the highest rate consistent with borrowers satisfying their reservation utilities. The concave loan demand function suggests that, in the first beginning stage of the loan application, the firms are not so sensitive to the change of loan interest rate, due to the amount of loan demand. In this period, the firms’ loan demand does not change a lot, if banks increase the loan interest rate. However, with the growth of the loan interest rate, the firms’ loan demand function becomes more and more sensitive. It will change a lot, compared to the beginning stage, even banks’ loan interest rate changes a little. We can see from the *Fig 1* that the slope of the loan demand function is decreasing, it is consistent with what we describe here.

Similar steps to the previous part, we can get the same optimal bank monitoring level:

$$q^{*}=\frac{r_{L}}{2c}$$ (3.1.12)

In the same market structure, banks can choose the loan interest rate by themselves, while the capital *k* is still determined by others, for example, the regulator. Similarly, if we want to find out the sign of $\frac{\partial q}{\partial r^{*}}$, we still need to figure out $\frac{\partial r_{L}}{\partial r^{*}}$. By substituting the optimal bank monitoring level q^*^, into the above bank’s expected profit function, we can get:

$$\Pi =\left[\frac{r_{L}}{2c}r_{L}-(1-k)\left(r^{*}+\alpha \right)-k\left(r^{*}+\beta \right)-\left(c{\left(\frac{r_{L}}{2c}\right)}^{2}+d\right)\right]\left(a-br_{L}^{2}\right)$$

$$=\left[\frac{r_{L}^{2}}{4c}-r^{*}-(1-k)\alpha -k\beta -d\right]\left(a-br_{L}^{2}\right)$$ (3.1.13)

Assuming $G=\frac{\partial \Pi }{\partial r_{L}}=0$, by using the Implicit Function Theorem, we can get the second order condition of bank’s expect profit respect to the loan interest rate:

$$\frac{\partial^{2}\Pi }{\partial r_{L}^{2}}=\frac{\partial G}{\partial r_{L}}=-\frac{3b}{c}r^{3}+\frac{a}{2c}\left[r^{*}+(1-k)\alpha +k\beta +d\right]$$ (3.1.14)

Therefore, in order to claim that the loan interest rate is chosen by the bank consistently with maximal profits, we need to keep the second order condition of bank profit respect to loan interest rate is strictly negative. It means $\frac{\partial^{2}\Pi }{\partial r_{L}^{2}}=\frac{\partial G}{\partial r_{L}}<0$.


$$\text{So},\text{we}\;\text{can}\;\text{get:}r_{L}>\sqrt{\frac{a}{6b}+\frac{2c}{3}\left[r^{*}+(1-k)\alpha +k\beta +d\right]},\text{if}\frac{\partial^{2}\Pi }{\partial r_{L}^{2}}=\frac{\partial G}{\partial r_{L}}<0.$$ (3.1.15)

$$\text{and},\;\frac{\partial G}{\partial r_{*}}=2br_{L}>0$$ (3.1.16)

$$\frac{\partial r_{L}}{\partial r^{*}}=-\frac{\partial G/\partial r^{*}}{\partial G/\partial r_{L}}>0$$ (3.1.17)

Due to the Eq *(3.1.5)*, $\frac{\partial q}{\partial r^{*}}$ is positively correlated with $\frac{\partial r_{L}}{\partial r^{*}}.$


$$\text{Similarly},\frac{\partial q}{\partial r^{*}}>0$$ (3.1.18)

This inequality implies that the relationship between the bank monitoring effort and risk-free interest rate is always positive. The higher risk-free rate, the higher a bank monitoring effort will be. Therefore, a lower policy rate will always increase bank risk-taking. So, we can obtain our result as follows:

**Proposition 2** *When the banks monitoring cost function is a convex function and the firms loan demand function is the assumed concave function*, *if banks can decide the size of loan interest rate*, *r*_*L*_*, banks will always set it in the interval of $\left(\sqrt{\frac{a}{6b}+\frac{2c}{3}\left[r^{*}+(1-k)\alpha +k\beta +d\right]},\sqrt{\frac{a}{b}}\right)$, banks monitoring increases with monetary policy rate, $\frac{\partial q}{\partial r^{*}}>0$Banks will take more risk if the monetary policy rate decreases.*

The intuition behind those two propositions is that a low monetary policy rate will result in a low loan interest rate, a low interest rate paid to debt and equity. Consequently, banks would reduce the valuations of their investments, pay low effort, and take more risk. This is explained from the value effect perspective, and for the search-for-yield effect, if the policy rate is low, banks will search for the projects with higher return, which generally come with higher risk. So, banks would like to grant loans to those projects with higher risk to get higher return, and the higher return will be used to pay the cost of debt and equity. Therefore, from banks perspective, the search-for-yield effect will occur at the stage of searching for loans, and the value effect will work at the stage of loan monitoring effort allocation.

*b. Piecewise loan demand function*. In the second case, a piecewise function, we can assume that the loan demand function is:

$$F\left(r_{L}\right)=\left\{\begin{array}{l} a,\text{if}\;r_{L}\leq r_{L}^{*}\\ 0,\text{if}\;r_{L}>r_{L}^{*} \end{array},\text{where}\;0<r_{L}^{*}<1\right.$$ (3.1.19)

**Fig 2:**
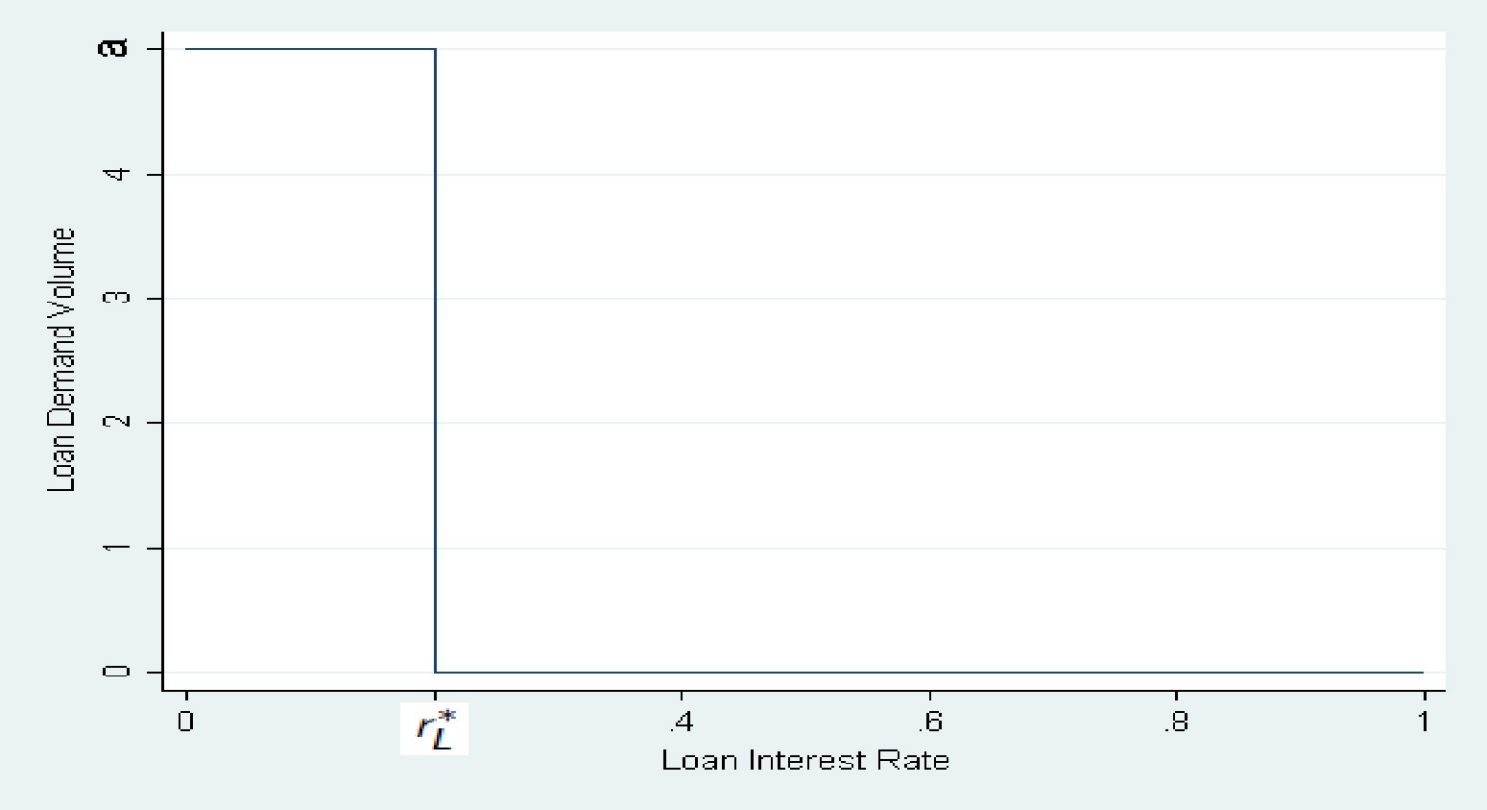
Piecewise loan demand function.

$r_{L}^{*}$can be interpreted as either the maximum return on projects, or as the highest rate consistent with borrowers satisfying their reservation utilities. In this case, firms will have a fixed loan demand, which is *a*, as long as $r_{L}\leq r_{L}^{*}$. This is because firms still need the loan from banks, as long as the loan interest rate is lower than their project’s return. There will be some profit for firms. So, their demand function will not change, if the banks do not charge more than their yield. This case can be shown in *Fig 2*.

Similarly, we can calculate the optimal bank monitoring effort according to this concave bank’s expected profit function in this case:

$$q^{*}=\frac{r_{L}^{*}}{2c}$$ (3.1.20)

So, q^*^ is consistent, and irrelevant with monetary policy rate. Banks will always choose the optimal loan interest rate level $r_{L}^{*}$, and the monitoring level $\frac{r_{L}^{*}}{2c}$. Then, we can obtain the result:

**Proposition 3** *When the banks monitoring cost function is a convex function and the firms loan demand function is the assumed piecewise function*, *banks will always choose a fixed optimal level of loan interest rate and a fixed optimal monitoring level*, *which is depended on the optimal loan interest rate. Banks risk-taking level is not affected by monetary policy rate*.

### 3.2 Theoretical model 2

In this part, we will introduce a similar theoretical model, while with the assumption of limited liability of banks in case of failure, to analyse if the relationship between monetary policy and bank risk-taking changes or not.

Except for the assumption of limited liability of banks in case of failure, all other assumptions in our model 2 are the same as those in model 1. Under those assumptions, the lenders are not liable for the debts if the borrowers fail to repay the loan. Therefore, our new banks’ expected profit function can be expressed as:

$$\Pi =\left[q\left(r_{L}-(1-k)r_{D}\right)-kr_{E}-C(q)\right]F\left(r_{L}\right)$$ (3.2.1)

*Where, Π is the bank’s expected profit*;

q is the monitoring effort, or the probability of the loan repayment;

r_L_ is the interest rate charged by banks;

k is the portion of bank assets financed with equity;

1*—k is the fraction of bank’s portfolio funded by deposits;*

r_D_ is the interest rate paid to depositors by bank;

r_E_ is the interest rate paid to equity investors by bank;

*C(q*) *is the cost function of bank monitoring*;

*F*(*r*_*L*_) *is the firms’ loan demand function*.

By substituting the expression functions of *r*_*D*_, *r*_*E*_, *C*(*q*), and *F*(*r*_*L*_) into the bank’s expected profit function, we can obtain a more detailed bank’s expected profit function:

$$\Pi =\left[qr_{L}-q(1-k)\left(r^{*}+\alpha \right)-k\left(r^{*}+\beta \right)-\left(cq^{2}+d\right)\right]\left(a-br_{L}\right)$$ (3.2.2)

We can see from the bank expected profit function, the profit per unit lent equals to the possible income of the loan (*qr*_*L*_), less the costs on deposit if the borrowers success to repay the loan (*q*(1—*k*)(r^*^+*α*)), takes off the cost of equity (*k*(r^*^+*β*)), and minus the cost of bank monitoring (*cq*^2^ + *d*). If a bank’s projects succeed, it will receive the payment from the loan and pay costs of debt, equity and bank monitoring. When a bank’s project fails, which means the borrower cannot return any repayment, the bank will receive no revenue, but, because of the limited liability, it does not need to pay the cost of debt either. Bank has to repay shareholders always, because the cost of equity is borne irrespective of the bank’s revenue. Because the above equation is a concave function of the bank’s monitoring effort, there exists a maximum value of the bank’s expected profits, and an optimal level of bank’s monitoring effort. We can get the optimal monitoring effort level, by calculating the function:

$$\frac{\partial \left[qr_{L}-q(1-k)\left(r^{*}+\alpha \right)-k\left(r^{*}+\beta \right)-\left(cq^{2}+d\right)\right]}{\partial q}\left(a-br_{L}\right)=0$$ (3.2.3)

Therefore, the optimal bank monitoring effort level will be:

$$q^{*}=min\left\{\frac{r_{L}-(1-k)\left(r^{*}+\alpha \right)}{2c},1\right\}$$ (3.2.4)

We assume the value of $\frac{r_{L}-(1-k)\left(r^{*}+\alpha \right)}{2c}$ will always be not larger than one. So, the optimal value of bank monitoring effort will equal to $\frac{r_{L}-(1-k)\left(r^{*}+\alpha \right)}{2c}$. The above function shows a more complicated relationship between bank’s optimal monitoring effort level, loan interest rate and risk-free interest rate. In the following part, we will discuss more detail about the relationship between bank monitoring effort level (bank risk-taking) and policy rate, when the lender is facing a convex monitoring cost function, and the borrower has different loan demand functions.

#### 3.2.1 Linear loan demand function

Taking the loan interest rate (*r*_*L*_) as given, the relationship between bank monitoring effort and risk-free interest rate is non-positive, because $\frac{\partial q^{*}}{\partial r^{*}}\leq 0$. But in most cases, bank can decide the loan interest rate by itself. So, we will now mainly consider the case, in which, bank can choose the level of loan interest rate. Similarly, to obtain the effect of monetary policy on bank risk-taking, we need to know the first order condition of bank optimal monitoring effort with respect to risk free rate, because the assumption that monetary policy can affect the cost of bank’s capital by changing the risk-free rate.


$$\frac{\partial q^{*}}{\partial r^{*}}=-\frac{\frac{\partial r_{L}}{\partial r^{*}}-(1-K)}{2c}$$ (3.2.5)

To obtain $\frac{\partial r_{L}}{\partial r^{*}}$, we can substitute the optimal bank optimal monitoring effort q^*^ into the bank’s expected profit function:

$$\Pi =\left[\frac{r_{L}-(1-k)\left(r^{*}+\alpha \right)}{2c}\left(r_{L}-(1-k)r_{D}\right)-kr_{E}-\left[c{\left(\frac{r_{L}-(1-k)\left(r^{*}+\alpha \right)}{2c}\right)}^{2}+d\right]\right]\left(a-br_{L}\right)$$

$$=\left[\frac{{\left(r_{L}-(1-k)\left(r^{*}+\alpha \right)\right)}^{2}}{4c}-k\left(r^{*}+\beta \right)-d\right]\left(a-br_{L}\right)$$ (3.2.6)

We assume $G=\frac{\partial \Pi }{\partial r_{L}}$, by using the Implicit Function Theorem, we can get:

$$\frac{\partial r_{L}}{\partial r^{*}}=-\frac{\partial G/\partial r^{*}}{\partial G/\partial r_{L}}$$ (3.2.7)

To achieve the maximum expected profit, bank has to set the level of loan interest rate to satisfy $\frac{\partial G}{\partial r_{L}}<0$. So, we just need to consider the sign of $\frac{\partial G}{\partial r^{*}}$:

$$\frac{\partial G}{\partial r^{*}}=-\frac{1-k}{2c}\left(a-br_{L}\right)-b\left[\frac{\left[r_{L-(1-k)\left(r^{*}+\alpha \right)}\right](k-1)}{2c}-k\right]$$ (3.2.8)

For $k\to 0,\frac{\partial G}{\partial r^{*}}<0$, as a result, $\frac{\partial r_{L}}{\partial r^{*}}<0$ as well. As $k\to 0,\frac{\partial q^{*}}{\partial r^{*}}=\frac{\frac{\partial r_{L}}{\partial r^{*}}-(1-k)}{2c}\to \frac{1}{2c}\left(\frac{\partial r_{L}}{\partial r^{*}}-1\right)<-1<0$. For $k\to 1,\frac{\partial G}{\partial r^{*}}>0$ as a result, $\frac{\partial r_{L}}{\partial r^{*}}>0$ as well. As $k\to 1,\frac{\partial q^{*}}{\partial r^{*}}=\frac{\frac{\partial r_{L}}{\partial r^{*}}-(1-k)}{2c}\to \frac{1}{2c}\left(\frac{\partial r_{L}}{\partial r^{*}}\right)>0$. Therefore, at one extreme of *k*, *k* → 0, the effect of monetary policy on bank monitoring effort is negative, while at the other extreme of *k*, *k* → 1, the effect is positive. This means there must exist a value k^*^∈(0,1), for any $k<k^{*},\frac{\partial q^{*}}{\partial r^{*}}>0$, and for any $k>k^{*},\frac{\partial q^{*}}{\partial r^{*}}>0$. It shows that, following a change in monetary policy, risk-taking can react in either direction depending on whether the bank finances with equity a high or a low fraction of its assets. According to this, we can obtain our next proposition:

**Proposition 4** *When banks monitoring cost function is a convex function and firms loan demand function is a linear function*, *if banks can decide the size of loan interest rate*, *r*_*L*_*, banks that financed with low portion of equity, will pay less monitoring effort with the increase of monetary policy, $\frac{\partial q^{*}}{\partial r^{*}}<0$. Banks will take more risk with the monetary policy increases. Banks who financed with high portion of equity, will pay more monitoring effort with the increase of monetary policy, $\frac{\partial q^{*}}{\partial r^{*}}>0$. Banks will take more risk with the monetary policy decreases.*

#### 3.2.2 Non-linear loan demand function

In this section, we will also test the effect of the alternative loan demand functions. We look at two different cases: first, the loan demand function is a concave function; while second, the loan demand function is a piecewise function.

*a. Concave loan demand function*. In the first case, we assume that the concave loan demand function is:

$$F\left(r_{L}\right)=a-br_{L}^{2},where,0<a\leq b,\;and\;0<r_{L}<\sqrt{\frac{a}{b}}.$$ (3.2.9)

Therefore, the new bank expected profit function is:

$$\Pi =\left[q\left[r_{L}-(1-k)\left(r^{*}+\alpha \right)\right]-k\left(r^{*}+\beta \right)-\left(cq^{2}+d\right)\right]\left(a-br_{L}^{2}\right)$$ (3.2.10)

By maximizing the bank’s expected profit, we can obtain the optimal bank monitoring level:

$$q^{*}=min\left\{\frac{r_{L}-(1-k)\left(r^{*}+\alpha \right)}{2c},1\right\}$$ (3.2.11)

In the same market structure, in order to find out the sign of $\frac{\partial q}{\partial r^{*}}$, we can figure out the sign of $\frac{\partial r_{L}}{\partial r^{*}}$ first. By substituting the optimal bank monitoring level q^*^ into the above bank’s expected profit function (3.2.10):

$$\Pi =\left[\frac{r_{L}-(1-k)\left(r^{*}+\alpha \right)}{2c}\left(r_{L}-(1-k)r_{D}\right)-kr_{E}-\left[c{\left(\frac{r_{L}-(1-k)\left(r^{*}+\alpha \right)}{2c}\right)}^{2}+d\right]\right]\left(a-br_{L}^{2}\right)$$

$$=\left[\frac{{\left(r_{L}-(1-k)\left(r^{*}+\alpha \right)\right)}^{2}}{4c}-k\left(r^{*}+\beta \right)-d\right]\left(a-br_{L}^{2}\right)$$ (3.2.12)

Assuming $G=\frac{\partial \Pi }{\partial r_{L}}$, according to the Implicit Function Theorem:

$$\frac{\partial r_{L}}{\partial r^{*}}=-\frac{\partial G/\partial r^{*}}{\partial G/\partial r_{L}}$$ (3.2.13)

To achieve the maximum expected profit, bank has to set the level of loan interest rate to satisfy $\frac{\partial G}{\partial r_{L}}<0$. So, we just need to consider the sign of $\frac{\partial G}{\partial r^{*}}$:

$$\frac{\partial G}{\partial r^{*}}=-\frac{1-k}{2c}\left(a-br_{L}\right)-2br_{L}\left[\frac{\left[r_{L-(1-k)\left(r^{*}+\alpha \right)}\right](k-1)}{2c}-k\right]$$ (3.2.14)

If $k\to 0,\frac{\partial G}{\partial r^{*}}<0$ it means, $\frac{\partial r_{L}}{\partial r^{*}}<0$ as well. When $k\to 0,\frac{\partial q^{*}}{\partial r^{*}}=\frac{\frac{\partial r_{L}}{\partial r^{*}}(1-k)}{2c}\to \frac{1}{2c}\left(\frac{\partial r_{L}}{\partial r^{*}}-1\right)<¯1<0$. While for $k\to 1,\frac{\partial G}{\partial r^{*}}>0$ as a result, $\frac{\partial r_{L}}{\partial r^{*}}>0$. As $k\to 1,\frac{\partial q^{*}}{\partial r^{*}}=\frac{\frac{\partial r_{L}}{\partial r^{*}}-(1-k)}{2c}\to \frac{1}{2c}\left(\frac{\partial r_{L}}{\partial r^{*}}\right)>0$. Therefore, at one extreme of *k*, *k* → 0, the effect of monetary policy on bank monitoring effort is negative, while at the other extreme of *k*, *k* → 1, the effect is positive. This means there must exist a value k^*^ ∈ (0,1), for any $k<k^{*},\frac{\partial q^{*}}{\partial r^{*}}<0$, and for any $k>k^{*},\frac{\partial q^{*}}{\partial r^{*}}>0$. According to this, we can obtain our proposition 5:

**Proposition 5** *When banks monitoring cost function is a convex function and firms loan demand function is the assumed concave function*, *if banks can decide the size of loan interest rate*, *r*_*L*_*, banks that financed with low portion of equity, will pay less monitoring effort with the increase of monetary policy, $\frac{\partial q^{*}}{\partial r^{*}}<0$. Banks will take more risk with the monetary policy increases. Banks who financed with high portion of equity, will pay more monitoring effort with the increase of monetary policy, $\frac{\partial q^{*}}{\partial r^{*}}>0$. Banks will take more risk with the monetary policy decreases.*

The intuition behind the proposition 4 and proposition 5 is that: in the case of limited liability for banks, when the policy rate is low, banks with high equity ratio need to search for higher yield projects to pay the cost of equity. That is because high equity ratio banks only finance themselves in a small percentage of debt, if there is not enough successful loan repayment, they are only not liable for those small part of the debt. Banks still need to pay for the equity cost, which takes a large percentage. This is explained from the search-for-yield effect perspective, while from the value effect perspective, a low monetary policy rate will result in a low loan interest rate, a low interest rate paid to debt and equity. Consequently, banks with higher equity ratios generally need a higher return. Therefore, they would reduce the valuations of their investments, which are in low interest rate, and banks will pay low effort, meanwhile, take more risk. Similarly, from the bank’s perspective, the search-for-yield effect will occur at the stage of searching for loans, and the value effect will work at the stage of loan monitoring effort allocation.

*b. Piecewise loan demand function*. In the second case, similar to the first theoretical model, we assume the new loan demand function is:

$$F\left(r_{L}\right)=\left\{\begin{array}{l} a,\;if\;r_{L}\leq r_{L}^{*}\\ 0,\;if\;r_{L}>r_{L}^{*} \end{array},\text{where}\;0<r_{L}^{*}<1\right.$$ (3.2.15)

$r_{L}^{*}$can be interpreted as either the maximum return on projects, or as the highest rate consistent with borrowers satisfying their reservation utilities. By substituting this new loan demand function into bank’s expected profit function:

$$\Pi =\left\{\begin{array}{c} \left[q\left[r_{L}-(1-k)\left(r^{*}+\alpha \right)\right]-k\left(r^{*}+\beta \right)-\left(cq^{2}+d\right)\right]a,\;if\;r_{L}\leq r_{L}^{*}\\ 0,\;if\;r_{L}>r_{L}^{*} \end{array}\right.$$ (3.2.16)

If firms have this kind of loan demand function, banks will always optimally set their loan interest rate at the level, $r_{L}^{*}$, to achieve the maximum profit. And the expected profit function will be:

$$\Pi =\left[q\left[r_{L}^{*}-(1-k)\left(r^{*}+\alpha \right)\right]-k\left(r^{*}+\beta \right)-\left(cq^{2}+d\right)\right]a$$ (3.2.17)

Similar to previous parts, the optimal bank monitoring effort is:

$$q^{*}=\frac{r_{L}^{*}-(1-k)\left(r^{*}+\alpha \right)}{2c}$$ (3.2.18)

Because the level of $r_{L}^{*}$, is fixed, bank optimal monitoring effort is unambiguously negatively correlated with the monetary policy rate. So, a higher monetary policy rate will reduce the level of bank optimal monitoring effort, which means a higher monetary policy rate will increase the level of bank risk-taking. According to this, we can obtain our final proposition:

**Proposition 6** *When banks monitoring cost function is a convex function and firms loan demand function is the assumed piecewise function*, *if banks can decide the size of loan interest rate*, *r*_*L*_, *banks will take more risk with the monetary policy rate increases*.

## 4 Empirical model

### 4.1 Methodology and data

In this section, we will use empirical regression to test and analyze the effect of monetary policy on bank risk-taking based on the above theoretical models. Our basic regression model is:

$$q_{i,t}=\alpha +\gamma \Pi_{i,t}+\delta r_{i,t}+\theta r_{t}^{*}+\rho k_{i,t}+u_{i}+\varepsilon_{i,t}$$ (4.1.1)

Where, q_i,t_ is risk-taking variable of bank i, in period t;

*Π_it_ is the profit of bank i*, *in period t*;

r_it_ is the loan interest rate charged by bank i, in period t;

$r_{t}^{*}$ is the risk-free rate in period t;

k_it_ is the portion of assets financed with equity in bank i;

u_i_ is the random effect specific to individual bank i;

ε_it_ is the error term.

All the variables in section 3 are included in this empirical model. Because we want to test the effect of monetary policy rate on bank risk-taking, we set the bank risk-taking variable at the left side of the function and set the policy rate variable as the main independent variable. Other independent variables include loan interest rate, equity ratio, bank profit, etc. These variables are nominal.

In this part, we use a unique database, which includes the balance sheet information (market values, ratios, etc.) for the listed top 30 banks by capitalization size over the period 2000 to 2017 in the United States. All the data involved in this paper are quarterly data, from quarter 1 in 2000 to quarter 1 in 2017. There are 1950 observations for all these 30 banks. Most bank relative data come from the Orbis BankFocus, and the policy rate data comes from Thomson Reuters DataStream.

Next, we will describe the variables in our model. We use Z-Score [37] as an inverse proxy of bank risk-taking level–dependent variable *q*_*i*,*t*_, in other words, it can be used as a proxy of bank monitoring effort level. It is a very common practice in existing literature [38–40]. There are also various other risk measures, but these often require market as well as accounting data and so may not be feasible in this case. Z-score can be used to measure the distance from insolvency [41], which is defined as a state where losses are larger than equity. A high Z-score means more stability in the bank. So, the higher Z-score is, the more monitoring effort will be paid by a bank, and the less risk will bank take. It can be calculated by the return on assets plus the equity to asset ratio divided by the standard deviation of return on assets. That is $Z=\frac{ROA+CAR}{\sigma (ROA)}$, where, ROA is the rate of return on assets (which is profit after tax over total equity), CAR is the capital assets ratio, and σ(*ROA*) is an estimate of the standard deviation of return on assets.

Intuitively, this measure of Z-score represents the number of standard deviations below the mean by which profits would have to fall so as to just deplete equity capital [42]. It has been widely used to measure the bank risk-taking level recently [39, 40]. Regarding the independent variables, we use banks’ net income to measure the banks’ profit (in a hundred thousand US Dollars). It is the banks’ profit after tax. In our database, this variable is calculated by profit before tax takes off the income tax expense, then plus the net profit for the year from discontinued operations. The loan interest rate (which is interest on loans over performing loans), equity ratio and policy rate are all measured in percentage. Because we are testing the effect of short-term interest rate on banks risk-taking, in this paper, the 3-month U.S. Treasury bill rate will be adopted to measure to risk free rate. For the data stationary, we will use the first difference of risk-free rate in our model.

Although all the variables have been included in the Eq (4.1.1), the empirical estimation of this model still presents a number of challenges. The two main identification limitations of testing the effect of monetary policy rate on bank risk-taking are the potential endogeneity of monetary policy rate to the risk-taking variable in the banking sector [2], and the persistence of bank risk-taking level [43]. Besides, the bank characteristics, such as profitability, bank equity ratio, loan interest rate, etc., may also be endogenous to the risk-taking level by banks. That is because causality may run in both directions–from left hand side variable to right hand variables and vice versa. Concerning the endogeneity of monetary policy to the bank risk-taking on the right-hand side of the equation, it is maybe particularly true during the period of the financial crisis, because there is a rapid expansion on the set of conventional and unconventional policy measure by central banks to improve the situation of banks and other financial institutions [44]. That means that central banks may have to change the monetary policy, especially the short-term interest rates, to affect banks’ risk-taking level, with the aim of stimulating the economy and stabilizing the financial market, in that period. For other bank specific controls, it is well-known that they can interact with the bank risk-taking variable, for example, the equity ratio can be regarded as a measure of bank capitalization, and a bank can change its capital structure by taking different levels of risk, which can change the investors’ investment strategy, according to their risk preference. On the other hand, a bank can also set different risk-taking targets by changing its capital structure, because “search for yield”. Hence, the bank can tradeoff higher levels of equity capital for risk assets, which indicates an endogenous relationship. From an econometric perspective, endogeneity implies that the interest rate and other bank specific variables are correlated with the error term, ε_*i*,*t*_, which may bias the estimation results. To tackle endogeneity, an instrumental variable method can be adopted, which will be introduced below.

The second main identification challenge in Eq (4.1.1) is the persistence of bank risk-taking. To deal with it, we will estimate a dynamic panel data model, which accounts for risk persistence. This means a lagged dependent variable (β*q_*i*,*t*-1_) can be added in the right-hand side of Eq (4.1.1) to build up this dynamic model. The coefficient *β* on the lagged risk variable may be viewed as the speed of adjustment from past risk-taking by banks. A value of *β* close to 0 means that the bank will accompany with a high speed to escape from the influence of its past risk-taking level, while a value of *β* close to 1 implies that the bank’s adjustment of past risk-taking is very slow. All the values between 0 and 1 suggest the presence of risk persists, and the past risk-taking level must have a positive influence on banks’ current risk-taking level. Therefore, the new equation that will be estimated in this stage has the following functional form:

$$q_{i,t}=\alpha +\beta q_{i,t-1}+\gamma \Pi_{i,t}+\delta r_{i,t}+\theta r_{t}^{*}+\rho k_{i,t}+u_{i}+\varepsilon_{i,t}$$ (4.1.2)

According to Delis and Kouretas [43], there are several theoretical reasons, which can be provided to explain the dynamic nature of bank risk term. First, persistence may reflect the existence of intense competition, which tends to increase the risk-taking of banks [45, 46]. Second, relationship-banking with risky borrowers will have a lasting effect on the levels of bank risk-taking, although banks will reduce the operating cost and improve efficiency, if they work with the same client. Thirdly, if the risk is associated with the business cycle, banks may spend more time to ease the effects of macroeconomic shock. Fourth, risks may persist due to regulation, in particular, banks may have to invest in risk assets over a long period of time, under some specific policy condition, for example, in the periods, when risk-free interest rate is low, or money supply is low, etc.

To obtain consistent and unbiased estimates of the relationship between monetary policy and bank risk-taking, the above Eq (4.1.2) can be estimated using the Generalised Method of Moments (GMM) for dynamic panel data. This model is introduced by Holtz-Eakin et al. [47] and Arellano and Bond [48] and further developed by Arellano and Bover [49] and Blundell and Bond [50]. Here, we choose the Arellano–Bond GMM estimator, that is because: first, it can be used to solve the endogeneity between risk-taking level and some of the right-hand side variables by means of appropriate instruments. Compared with the instrumental variable estimation—two-stage least squares or 2SLS, in the Arellano–Bond GMM estimator model, not only use the exogenous listed instruments, it also adds lagged levels of other endogenous regressors. This makes the endogenous variables pre-determined, and as a result, they will not be correlated with the error term in Eq (4.1.1) [51]. Second, the presence of the lagged dependent variable *q*_*i*,*t*-1_ on the right-hand side may give rise to autocorrelation. It will render the Ordinary Least Squares estimator biased and inconsistent even if the idiosyncratic errors are not serially correlated [52]. Hence, to solve it, the first-differenced lagged dependent variable will be used in the Arellano–Bond GMM estimator model.

As shown by Blundell and Bond [50, 53], the Arellano–Bond difference GMM estimator is less efficient than the system GMM, when the autoregressive parameter is high, and the time-series dimension of the underlying data is moderately small. This means that the lagged values of the variables are only weakly correlated with the endogenous variables and are weak instruments [54]. The first-differences GMM estimation also suffers from a loss of valuable observations. Under these conditions, the first-differences GMM estimation is likely to perform poorly. Hence, we will adopt the system GMM estimator suggested by Arellano and Bover [49], Blundell and Bond [50], because it is more plausible.

The system GMM estimator uses a system of two sets of equations: one differenced and one in levels. To be more specific, it combines the standard set of equations in first differences and an additional set of level equations. The system of equation takes the following form:

$$\left\{\begin{array}{l} q_{i,t}=\alpha +\beta q_{i,t-1}+\gamma \Pi_{i,t}+\delta r_{i,t}+\theta r_{t}^{*}+\rho k_{i,t}+u_{i}+\varepsilon_{i,t}\\ \Delta q_{i,t}=\alpha +\beta \Delta q_{i,t-1}+\gamma {\Delta \Pi }_{i,t}+\delta \Delta r_{i,t}+\theta \Delta r_{t}^{*}+\rho \Delta k_{i,t}+\varepsilon_{i,t}^{\prime } \end{array}\right.$$ (4.1.3 and 4.1.4)

Before talking about the empirical estimation results, we will have a look at the summary statistics and correlation coefficients between these variables. *Table 1* shows the descriptive statistics of all the unbalanced panel data in this model. The mean of Z-score is 0.1839, with a standard deviation of 0.0966. Because Z-score is used to describe the probability of bank succeeds, the highest success probability is around 0.5, while the lowest is 0.003. The average loan interest rate in the last 17 years is 0.03% higher than the average 3-month U.S. treasury bill rate. The equity ratio in U.S. top 30 banks varies from 4.54 to 54.03 in the last 17 years, which is a big range. So, we will have a look at the different performances of banks risk-taking decisions for banks with high equity ratio and low equity ratio.

**Table 1:** Descriptive statistics.

Variable	Observation	Mean	Standard Deviation	Min	Max
Z-score	1,950	0.1839292	0.0965261	0.0025459	0.4920304
Risk-free interest rate	1,950	1.419405	1.76845	-0.01	6.21
Loan interest rate	1,950	1.443896	0.5958023	-3.671252	5.405406
Equity ratio	1,950	10.89607	4.425616	4.54	54.03
Profit	1,950	8.106195	15.6693	-180	77.8

Z-Score is an inverse proxy of bank risk-taking level

Loan interest rate, equity ratio and risk-free interest rate are all measured in percentage

Risk-free interest rate is measured by 3-month U.S. treasury bill rate.

Source of data: Orbis BankFocus and Thomson Reuters DataStream

*Table 2* reports the correlation coefficients between all explanatory variables. It suggests that only the correlation between the interest rate variables (loan interest rate and risk free interest rate) is moderate, but still at an acceptable level. The result shows that multicollinearity will be unlikely to affect the following estimates. In what follows, some further empirical estimations will be discussed.

**Table 2:** Correlation matrix.

	Risk-free interest rate	Loan interest rate	Equity ratio	Profit
Risk-free interest rate	1			
Loan interest rate	0.4517	1		
Equity ratio	-0.1537	0.1306	1	
Profit	-0.0079	0.0578	-0.0794	1

Source of data: Orbis BankFocus and Thomson Reuters DataStream

**Fig 3:**
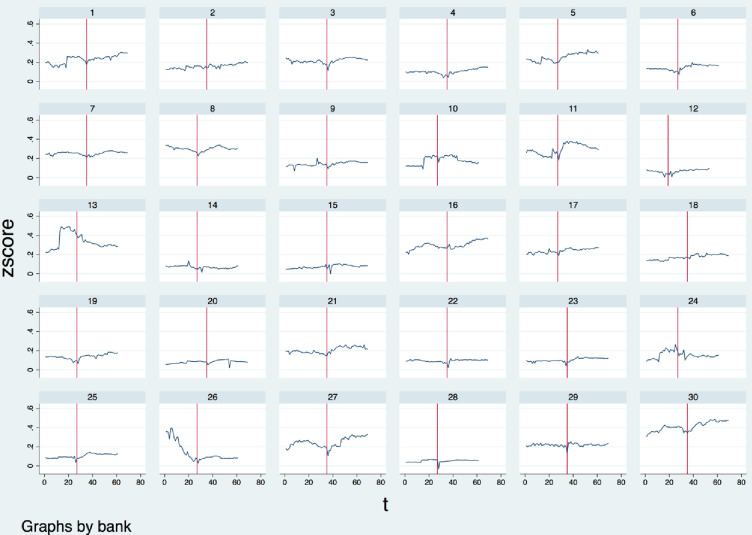
Individual bank time series values of Z-score. Vertical line at 2008 Q3. Source of data: Orbis BankFocus and Thomson Reuters DataStream.

**Fig 4:**
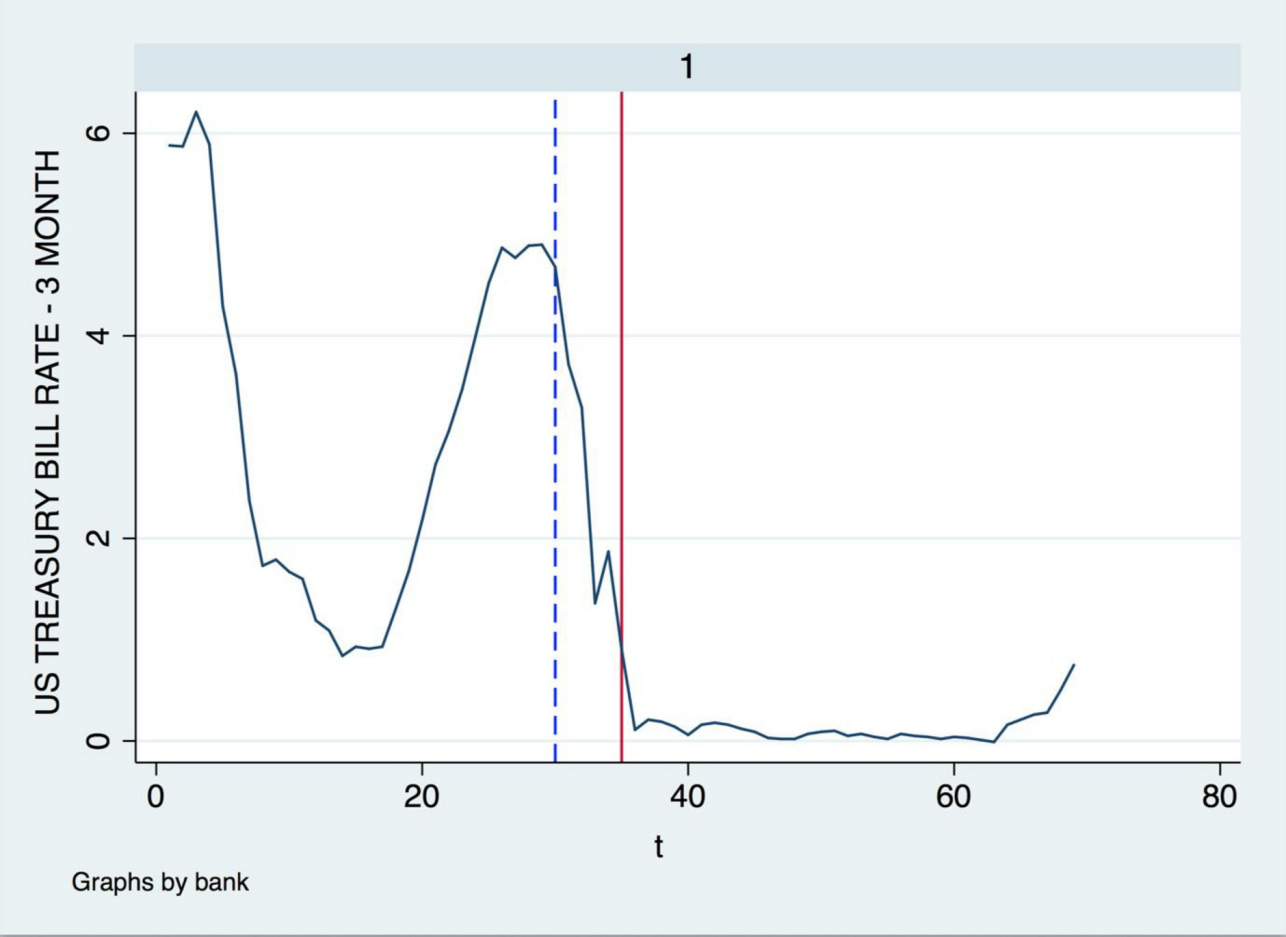
U.S. short-term treasury bill rate. Solid vertical line at 2008 Q3, dashed vertical line at 2007 Q2, Source of data: Orbis BankFocus and Thomson Reuters DataStream.

To find out if the financial crisis changed the banks’ risk attitude, in *Fig 3*, we compare the trend of their monitoring effort in the past 17 years first, especially during the period of the financial crisis. We set t = 35, which is the third quarter in 2008, during the period of financial crisis, and from the following figures, we find most banks had a significant drop of the Z-score value in 2008. That means, from this period, lots of banks relaxed the monitoring, and took more risk. This is consistent with the fact that around the period of 2008, lots of banks took on excessive risk, and eventually led to the financial crisis. Moreover, most banks loan portfolio monitoring levels had an increasing trend after the recent financial crisis. This can be explained that after the crisis, bankers and policymakers paid more attention to the risk-taking and be more enthusiastic to monitor banks’ loan portfolios, to avoid excessive risk-taking. In *Fig 4*, we set the same time for the change of the monetary policy rate, which is the solid vertical line. We also add a dashed vertical line in *Fig 4*, which is 2007 quarter 2. It is the time when the short-term interest rate started to drop. Between 2007 quarter 2 and the recent financial crisis, there is a one-year gap, in which, the bad outcome occurred. During that period, banks released their lending standard, took more and more risks, and finally, the financial crisis occurred. It is obvious that in the year 2008, the U.S. short-term treasurer bill rate dropped a lot. Thinking about the change in banks’ risk-taking level during the same period, there must be some interaction between the monetary policy rate and bank risk-taking during the financial crisis, Therefore, in the following section, we will discuss the influence of the financial crisis on bank risk-taking behavior for further detail.

### 4.2 Empirical results

Here, we present the main results of our empirical models. And depending on these results, we will conclude the relationship between bank risk-taking and monetary policy rate. we will also do some other tests to analyze bank risk-taking behavior. Let us start with results of our main estimations—Arellano-Bond dynamic panel data estimation, which is sectionalized into different periods.

#### 4.2.1 Main estimations

Following Roodman [51], to avoid instruments proliferation, we use a collapsed instrument matrix. It can help to create one instrument for every variable and lag distance. Otherwise, considerably more instrument variables will be created for each period of time, variable, and lag distance. On the other side of the number of instruments, the Hansen test may be weakened by too many instruments, which will affect the instruments’ validity. Therefore, in our results, the number of instruments is restricted by the collapsed instrument matrix to be 30, which is not larger than the number of groups. Moreover, we also use the dynamic panel-data estimation, with two-step system GMM, in which, the standard covariance matrix is robust to panel-specific autocorrelation and heteroskedasticity. The Arellano-Bond tests the first order autocorrelation, AR1, in the first difference errors. The result accepts the presence of the first order serial correlation, which means that the first lag of the dependent variable is exogenous. But the result rejects the presence of the second order serial correlation, AR2. Meanwhile, the Hansen test of overidentifying restrictions indicates that our instruments variables set is valid.

The *Table 3* presents the results of our main empirical estimations. It reports the coefficients, standard errors which are in parentheses, and its p-values which are indicated by *. In the first column, the full period observations are included, from the first quarter in 2000 to the first quarter in 2017, which involves the periods pre-crisis and post-crisis. In the second column, only the data before the financial crisis are analyzed. It contains the period from quarter 1 in 2000 to quarter 3 in 2008. Similarly, we test the observations after quarter 3 in 2008 as a post-crisis analysis, in the last column. The dependent variable is the inverse risk proxy—Z-score. In the following section, we will also test the effect of monetary policy on bank risk-taking by adopting other bank risk-taking measures for robustness checks. Similarly, another measure of interest rates will also be compared to test the robustness, because there is a fixed variable for short term interest rates used in this stage of estimations. The Wald-test and its associated p-value denote the goodness of fit of the regressions, AR1 and AR2 are the tests for first and second-order autocorrelation.

**Table 3:** Short-term interest rate and bank risk-taking: Arellano-Bond dynamic panel GMM estimators.

	2000–2017	2000–2008	2008–2017
Lagged inverse bank risk	0.412***	0.411***	0.524***
	(0.010)	(0.009)	(0.029)
Risk-free interest rate	0.002***	0.003***	0.011***
	(0.000)	(0.001)	(0.003)
Profit	0.0005***	0.001***	0.001***
	(0.000)	(0.000)	(0.000)
Loan interest rate	0.014***	-0.003***	0.003*
	(0.001)	(0.001)	(0.001)
Equity ratio	0.007***	0.008***	0.008***
	(0.000)	(0.000)	(0.001)
T	0.0005***	0.0002***	0.0002***
	(0.000)	(0.000)	(0.000)
No. of observations	1920	1020	870
No. of Instruments	30	30	30
No. of Banks	30	30	30
Wald-test	53616.330	66173.920	45272.140
Wald-test p-value	0.000	0.000	0.000
AR(1)	0.000	0.000	0.001
AR(2)	0.112	0.351	0.111
Hansen	0.218	0.554	0.277
Difference-in-Hansen tests (Hansen p-value)	0.175	0.332	0.230
Difference-in-Hansen tests (difference)	0.484	0.930	0489

* indicate statistical significance at the 10%

** indicate statistical significance at the 5%, and

*** indicate statistical significance at the 1%. Standard errors are in parentheses.

Following the results in the first column, it is obvious that the coefficients of all the variables are positive and strongly significant. It indicates all the explanatory variables have a positive influence on the inverse bank risk proxy–Z-score. in other words, these explanatory variables can affect the bank risk-taking behavior in a negative way. For the key variable–risk free interest rate, the positive and significant coefficient suggests that there is very strong evidence lower short-term risk-free interest rate will reduce the bank monitoring effort level, and encourage bank risk-taking significantly. With every percentage point decrease in the monetary policy rate, banks will take 0.002 percentage more risk. The profit and equity ratio also have a positive effect on bank monitoring effort level with the strength of 0.0005 and 0.007 respectively. This means that banks with less profit and low equity ratio would take more risk. This is because banks with less profit or low equity ratio will be more incentive to search higher return by taking more risk. A significantly positive regression also displays between the loan interest rate and inverse bank risk, with a coefficient of 0.014. It implies that banks with lower loan interest rates may seek higher risk. That is because, for a bank with a low loan interest rate, it will have a low loan interest revenue. This will in turn spur banks to invest in high risk assets to get high yield, and as a result, it will take more risk.

From *Table 3*, we can also conclude that bank risk-taking is persistent. There is strong evidence that the last period’s dependent variable has a significant positive effect on its current risk choice, with the coefficient 0.412. This means that banks’ last year risk-taking preference will have a positive effect on the current year’s risk-taking decision, compared with that in the year before last year, and this effect of last year will eventually dissipate.

We also compare the second and the third regressions, which are the periods before and after the 2008 financial crisis. Similar to the result in regression I, both last period inverse bank risk and risk-free interest rate have a positive effect on current bank risk-taking. However, the strength of these effects is stronger after the financial crisis, because the coefficient after the crisis of lagged inverse bank risk (0.524) is greater than that before the crisis (0.411), and the coefficient after the crisis of risk-free interest rate (0.011) is also larger than that before the crisis (0.003). This means with every percentage point decrease in risk-free rate, banks will take more risk after the crisis compared with that before the crisis, and bank last period risk-taking preference will affect more current risk behavior of banks. The sign of the loan interest rate’s coefficient has changed in the table. Banks take different strategies to take risk, when they consider loan interest rate only. This might be caused by some new regulations proposed after the crisis. In the following parts, we will consider a dummy variables estimation, to check the different risk-taking attitudes by different banks.

#### 4.2.2 Dummy variables estimation

Here, we compare the effects of monetary policy rate on bank risk-taking with different bank characteristics, such as, big banks and small size banks, high equity ratio banks and low equity ratio banks, and we also test the effect of monetary policy rate on bank risk-taking by adding the quarter variable into our model to check if the bank risk-taking behavior is different in different quarters. All these analyses are operated via dummy variables. The following *Table 4* shows the results of dummy variables in the Arellano-Bond dynamic panel model.

**Table 4:** Arellano-Bond dynamic panel model with dummy variables.

	2000–2017	2000–2008	2008–2017
Lagged inverse bank risk	0.175***	0.142***	0.257***
	(0.049)	(0.020)	(0.063)
Risk-free interest rate	0.004**	0.004***	-0.004***
	(0.002)	(0.001)	(0.003)
Profit	0.0003***	0.001***	0.001***
	(0.000)	(0.000)	(0.000)
Loan interest rate	0.007**	0.010***	0.017***
	(0.003)	(0.002)	(0.004)
Equity ratio	0.005***	0.007***	0.004*
	(0.001)	(0.000)	(0.002)
Post-crisis	-0.041***		
	(0.009)		
Big cap bank	0.132***	0.106***	-0.060***
	(0.015)	(0.018)	(0.007)
High equity ratio	0.086***	0.057***	0.038***
	(0.016)	(0.012)	(0.006)
Quarter 2	-0.001	-0.006***	0.001
	(0.002)	(0.001)	(0.001)
Quarter 3	0.002	-0.009***	0.002***
	(0.002)	(0.001)	(0.001)
Quarter 4	0.002	-0.004***	-0.003***
	(0.003)	(0.001)	(0.001)
No. of observations	1920	1020	870
No. of Instruments	30	30	30
No. of Banks	30	30	30
Wald-test	2681.510	37351.290	74121230
Wald-test p-value	0.000	0.000	0.000
AR(1)	0.000	0.000	0.000
AR(2)	0.250	0.711	0.024
Hansen	0.692	0.569	0.384
Difference-in-Hansen tests (Hansen p-value)	0.919	0.648	0.252
Difference-in-Hansen tests (difference)	0.151	0.324	0.683

The three regressions perform the estimation with period from Q1 2000 to Q1 2017, Q1 2000 to Q3 2008, and Q4 2008 to Q1 2017, respectively.

* indicate statistical significance at the 10%

** indicate statistical significance at the 5%, and

*** indicate statistical significance at the 1%. Standard errors are in parentheses.

Alongside the basic Arellano-Bond dynamic panel data estimation, we run a dummy estimation again which contains the post-crisis dummy, big capitalization banks dummy, high equity ratio dummy, and seasonal dummy. For the post-crisis dummy, we set the period after the financial crisis equal to 1 and the period before the financial crisis equal to 0. We rank these 30 banks by the size of capitalization and set the first 15 biggest banks to take the value 1, and others to be 0. For the equity ratio dummy, we divide these 30 banks into two groups. The group containing banks with equity ratio below the median equity ratio have the value 0, and the other group containing the banks with equity ratio above the median equity ratio take the value 1.

From the *Table 4*, we draw the following conclusions: the signs of right-hand side variable coefficients are all the same with the previous Arellano-Bond dynamic panel data estimation (in the third regression, the short-term interest rate has a negative sign coefficient which is not the same as the results in *Table 3*. However, it has a large P-value, which means the result of its coefficient is not significant). The results in the first and second regressions suggest there is a significant positive effect of risk-free rate on bank monitoring effort level. However, this effect is stronger than the previous estimation (the coefficients in *Table 4* are 0.004 in both regressions while the coefficients in *Table 3* of the risk-free interest rate are 0.002 and 0.003 respectively). After adding the dummy variables into the models, the lagged inverse bank risk receives low coefficients. The dummy variables do not change the effect of monetary policy on bank risk-taking. There is still significant evidence that short term interest rate has a positive effect on bank monitoring effort, and therefore, a negative effect on bank risk-taking.

Regarding the dummy variables themselves, the crisis reduces the bank’s risk-taking level by 0.041 units. And for the capitalization dummy variable, before the crisis, the banks with big sizes in capitalization are more sensitive to the change of risk-free interest. For every unit decrease in the policy rate, big size banks will take more risk, compared with the small size banks. However, after the crisis, the effect of monetary policy on bank risk-taking changes. the banks with small size in capitalization are more sensitive to the change of risk-free interest. But in the whole period of our sample, the case is still the same as that before the crisis. Therefore, the general effect of short-term interest rates on bank risk-taking is less pronounced for poorly capitalized banks. This can be explained by the “too big to fail” theory. About the equity dummy, the effect of short-term interest rates on bank risk-taking is stronger for the banks financed with a higher portion of equity. And this kind of effect has been reduced since the financial crisis. We also involve the seasonal dummy variables to test if the bank’s risk-taking differs over seasons. The results suggest that the seasonal dummy variables will change bank risk-taking levels, before the crisis. Season is still an important factor, but less important than that in the period before the crisis. For the whole period data, the seasonal dummies appear insignificant, because of the high level of P-value. However, before the crisis, banks would take less risk in the first quarter, compared with other quarters. This may be because, at the beginning of every year, banks will start their new project, but they have less stress to get all the targets finished. Compared with other quarters, the quarter near the end of the year might spur banks more to take risk to get the return. This is the reason why in quarter 3, before the crisis, the bank risk-taking level is higher than that in other quarters.

#### 4.2.3 Other measures of bank risk-taking and interest rate

To evaluate the robustness of our findings, we perform a different proxy of bank risk-taking variable and policy rate. First, we adopt the Non-Performing Loan (NPL) ratio to replace the Z-score. NPL ratio is a direct proxy to measure bank risk. It can be also regarded as a proxy for credit risk, while the Z-score is a measure of bank insolvency risk. NPL ratio can be calculated by the ratio of Non-Performing Loans to total loans. It reflects the quality of bank assets. Therefore, a high Non-Performing Loan ratio is associated with high credit risk. The effect of monetary policy on Non-Performing Loan ratio should be intuitively opposite, compared with the effect of monetary policy on Z-score. For the short-term interest rate, we use the federal funds rate to proxy the monetary policy rate. The following results from *Table 5* show the estimations of robustness tests. The coefficients on our main variables are qualitatively consistent and changing the proxies does not change the effect of monetary policy on bank risk-taking.

**Table 5:** Arellano-Bond dynamic panel model with other proxies of main variables.

	I	II	III	IV
Lagged inverse bank risk	0.412***	0.453***		
	(0.010)	(0.012)		
Lagged Non-Performing Loan ratio			1.055***	1.054***
			(0.003)	(0.004)
3-month U.S. treasury bill rate	0.002***		-0.012***	
	(0.000)		(0.002)	
Federal funds rate		0.001***		-0.019***
		(0.0002)		(0.004)
Profit	0.0005***	0.0005***	-0.008***	-0.008***
	(0.000)	(0.000)	(0.000)	(0.000)
Loan interest rate	0.014***	0.008**	0.155***	0.057***
	(0.001)	(0.000)	(0.004)	(0.005)
Equity ratio	0.007***	0.009***	-0.020***	-0.023***
	(0.000)	(0.000)	(0.001)	(0.001)
No. of observations	1920	1920	1920	1920
No. of Instruments	30	30	30	30
No. of Banks	30	30	30	30
Wald-test	53616.330	62398.010	1990000	96920.41
Wald-test p-value	0.000	0.000	0.000	0.000
AR(1)	0.000	0.000	0.000	0.000
AR(2)	0.112	0.258	0.763	0.762
Hansen	0.218	0.300	0.259	0.263
Difference-in-Hansen tests (Hansen p-value)	0.175	0.239	0.148	0.124
Difference-in-Hansen tests (difference)	0.484	0.534	0.768	0.898

The four regressions perform the estimation with: Z-score and 3-month U.S. treasury bill rate as the main variables in regression I, Z-score and federal funds rate as the main variables in regression II, NPL and 3-month U.S. treasury bill rate as the main variables in regression III, NPL and federal funds rate as the main variables in regression IV.

* indicate statistical significance at the 10%

** indicate statistical significance at the 5%, and

*** indicate statistical significance at the 1%. Standard errors are in parentheses.

## 5 Conclusion

In this paper, we identify the impact of the monetary policy on the behaviour of banks risk-taking in both theoretical and empirical foundations. In our models, monetary policy may affect banks’ perceptions of risk-taking, and their attitude to risk-taking in three forces: the value effect, the search-for-yield effect and the risk-taking-channel effect.

In the theoretical analysis, we compare several cases in which different market structures, reasonable bank monitoring cost functions and different loan demand functions exist. By letting banks maximize their profits and achieve equilibrium, we have the theoretical results of our model, with the assumption of unlimited liability for the bank. Firstly, for a convex monitoring cost function, and a linear loan demand function, when the capital structure is exogenous, and banks can choose loan interest rate, a lower risk-free rate unambiguously reduces monitoring and, hence, unambiguously increases bank risk-taking. Secondly, for a convex monitoring cost function, and the assumed concave loan demand function, when the capital structure is exogenous, banks can also choose loan interest rate, the result is similar that banks monitoring increases with monetary policy rate. Banks will take more risk with the monetary policy rate decreases. Finally, for a convex monitoring cost function, and the assumed piecewise loan demand function, when the capital structure is exogenous, bank’s risk-taking level is fixed according to the optimal loan interest rate and it is not affected by monetary policy rate.

Some different results exist when we include the assumption of limited liability for the bank in the case of failure. Firstly, for a convex monitoring cost function, if a bank finances itself with a high equity ratio, both models with linear loan demand function and the assumed concave loan demand function, can predict a positive correlation between the risk-free interest rate and bank monitoring effort. Therefore, they can also predict a negative correlation between the policy rate and bank risk-taking. A low policy rate level will increase bank risk-taking, and a high policy rate level will reduce bank risk-taking. Secondly, for a convex monitoring cost function, the model with the assumed piecewise loan demand function, can predict a negative correlation between risk free interest rate and bank monitoring effort. Banks will take more risk with the monetary policy rate increases.

Regarding the empirical analysis, this paper provides strong and wide evidence, and shows that a low short-term interest rate can contribute to the increase of bank risk-taking. Banks will take more risk if the monetary policy rate decreases. Moreover, bank risk-taking behavior can be affected by lots of factors, such as bank’s capitalization, equity ratio, season, etc. The bank risk-taking level had been reduced by the recent financial crisis, and banks take less risk after the crisis, than that in the period of pre-crisis. Meanwhile, regarding bank’s characteristics, the effect of short-term interest rates on bank risk-taking is less pronounced for poorly capitalized banks; the effect of short-term interest rates on bank risk-taking is also stronger for the banks financed with a higher portion of equity. Finally, seasons will change bank risk-taking behavior as well, and power is especially strong in the period of pre-crisis.

Even though banks have increasingly more effort to control risk-taking after the recent financial crisis, the risk-free interest rate has been still at a very low level since the financial crisis. So, for bankers and policymakers, it is even more important to monitor the banks’ risk-taking level regularly and frequently.
